# A Rare Case of Jejunal Pseudoaneurysm Presenting as Acute Small Bowel Obstruction After Blunt Trauma: Discussion, Management Dilemmas, and a Review of Relevant Literature

**DOI:** 10.7759/cureus.5655

**Published:** 2019-09-14

**Authors:** Manick Saran, Saptarshi Biswas

**Affiliations:** 1 Miscellaneous, Lake Erie College of Osteopathic Medicine, Erie, USA; 2 Trauma Surgery, Allegheny Health Network, Pittsburgh, USA

**Keywords:** pseudo-aneurysm

## Abstract

Visceral artery aneurysms (VAAs) and visceral artery pseudoaneurysms (VAPAs) are often found incidentally through imaging of a patient presenting with vague symptoms of abdominal pain, hematochezia, hematemesis, and melena. Due to the asymptomatic nature, the etiology is often unknown. However, suspicion for VAA and VAPA should remain high for those presenting with symptoms listed above after trauma or pancreatitis. Here we discuss a case of traumatic ileocolic pseudoaneurysm that has only been discussed a handful of times in the literature.

## Introduction

Aneurysms or pseudoaneurysms occurring in visceral arteries and their branches such as the celiac trunk, superior mesenteric artery, and inferior mesenteric artery are termed either visceral aneurysms (VAAs) or visceral pseudoaneurysms (VAPAs). VAA and VAPA of the splanchnic arteries is an uncommon entity with the incidence ranging from 0.1% to 2% of people [1]. The most common splanchnic artery affected by VAA or VAPA is the splenic artery, followed by hepatic arteries, celiac trunk, and the superior mesenteric artery [1]. The other smaller splanchnic arteries are less common. In recent years, the incidence may have increased due to better imaging modalities. Pseudoaneurysms and aneurysms are caused by a variety of different etiologies including iatrogenic causes, trauma, injury by a tumor, infection, vasculitis, inflammation, and collagen vascular diseases such as Ehlers-Danlos syndrome [2]. VAAs and VAPAs are most commonly found as incidental findings on CT and do not often present with any clinical symptoms. Due to their rarity, it is important to discuss VAPAs and their clinical workup. Here we present the case of an 80-year-old male who presented with a relatively rare case of an ileocolic pseudoaneurysm that has only been discussed in the literature nine times before.

## Case presentation

An 80-year-old male presented to the ED with a chief complaint of only vague abdominal pain after suffering a fall off a ladder at home. He stated that the pain came on acutely, intermittent and colicky and severe in intensity with bouts of acute exacerbations accompanied by significant nausea and vomiting. Trauma surgery was consulted and he was worked up in the routine advance trauma life support (ATLS) protocol. Primary and secondary were essentially negative except for focal abdominal tenderness. His abdominal exam was significant for distension, upper abdominal tenderness to palpation, localized guarding, and no rigidity or masses.

He denied any loss of consciousness, chest pain, numbness, tingling, hematochezia, and hematemesis. Upon examination, his vital signs were notable for a temperature of 97.6F, pulse of 86 beats per minute, and blood pressure of 167/84. His abdominal exam was significant for distension, upper abdominal tenderness to palpation, guarding, and no masses. Labs were significant for glucose of 278, lactic acid of 3.5, hemoglobin of 13.6, and hematocrit of 38.7.

His past medical history includes atrial fibrillation, diabetes mellitus, essential tremor, hypertension, seizures, sleep apnea, and a thyroid disorder.

**Figure 1:**
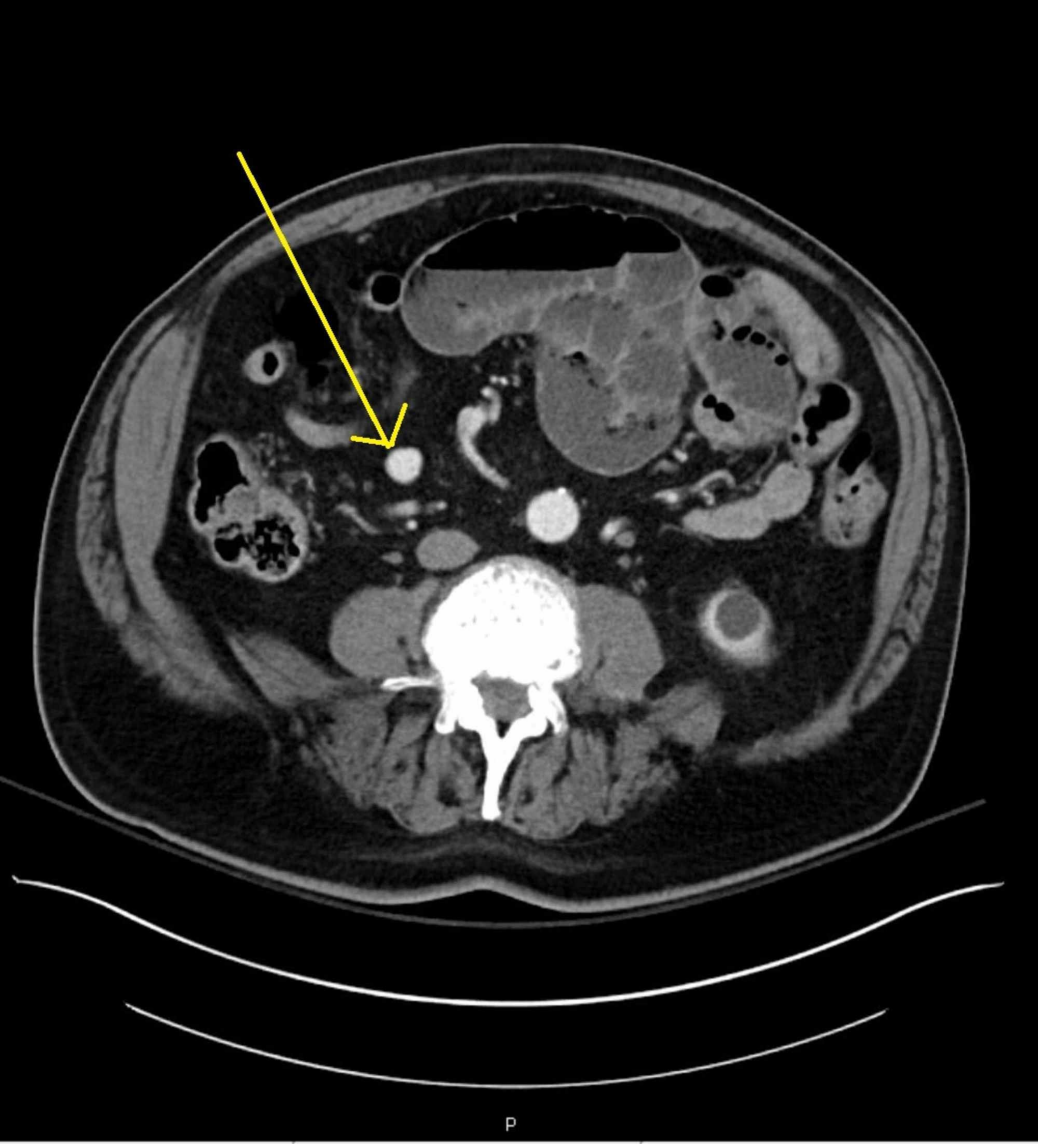
Pseudoaneurysm as seen on CT of the abdomen. Yellow arrow points to pseudoaneurysm.

Initial imaging at that time included a CT scan without contrast of the chest, abdomen, and pelvis. Significant findings on the CT showed few segmentally dilated loops of small bowel in the abdomen with air-fluid levels with a transition point. The findings suggested a small bowel obstruction as the bowel distally was more decompressed and normal caliber. He was diagnosed with a small bowel obstruction and admitted to the hospital. A nasogastric (NG) tube was placed; he was nil per os (NPO) and started on IV fluids. Serial abdominal examinations every six hours were ordered. A repeat CT scan with contrast was done which corroborated the earlier findings of a small bowel obstruction. However, in addition to the small bowel obstruction, findings on CT scan also included the incidental finding of a 1.6 cm x 1.4 cm rounded mesenteric structure in the right lower quadrant seen in Figure 1. This structure was isodense to contrast in adjacent arteries on the arterial phase which favored the diagnosis of a pseudoaneurysm over the diagnosis of an aneurysm. At this time, vascular surgery was consulted regarding the possible pseudoaneurysm.

Vascular surgery agreed with the findings of the possible pseudoaneurysm and recommended a mesenteric angiogram and possible endovascular coil embolization due to the high risk of rupture. The symptoms improved with conservative management and the patient started passing flatus and stool on hospital day three. The NG tube was removed and his diet was advanced as tolerated. Due to the resolution of his small bowel obstruction, the patient opted to go home and agreed to elective angiogram by IR the same week. He was discharged home on short-term enoxaparin.

**Figure 2:**
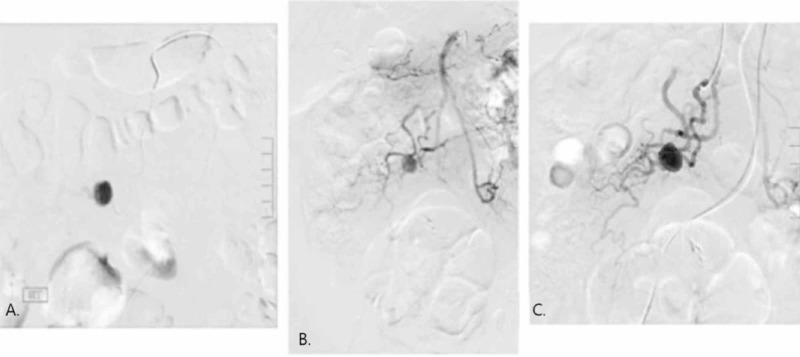
Angiogram of pseudoaneurysm. A. Microcatheter placed into pseudoaneurysm sac. B. Aneurysm with superior mesenteric artery injection. C. Aneurysm with ileal artery branch injection.

Later that week, the patient was referred for a diagnostic angiography to allow determination of an appropriate treatment strategy. At the time of the procedure, access was established from the right common femoral artery under ultrasound guidance. A 5 French sheath was placed. An abdominal aortagram was obtained after which selective catheterization of the superior mesenteric artery was performed. Arising from the ileocolic artery was the known 1.7 cm aneurysm, into which a microcatheter was placed and can be seen in Figure 2A. In Figure 2B,C the aneurysm can be seen with a superior mesenteric artery and an ileal artery branch injection, respectively. Super selective catheterization demonstrated a single feeding and draining artery to the aneurysm with no collateral filling. The patient elected to defer treatment at that time planning to discuss with his primary care physician (PCP) the benefits of embolization or waiting and watching. The patient saw his PCP a month later and at that time decided to proceed with embolization at another facility.

**Figure 3:**
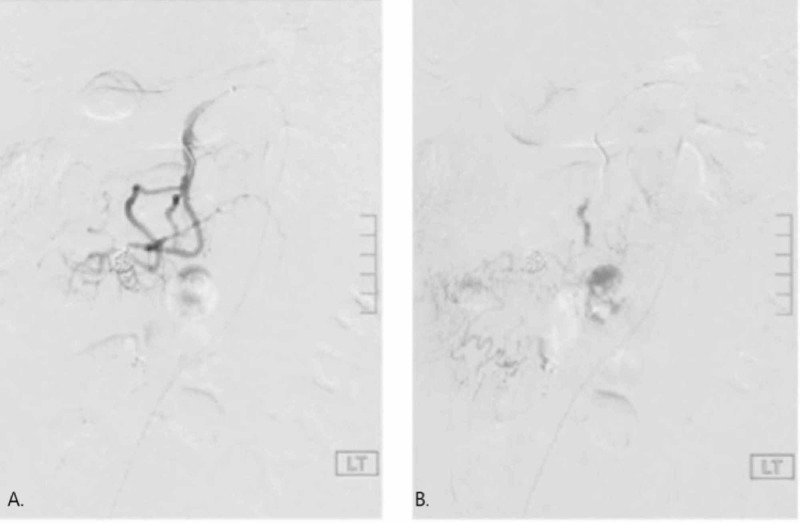
Embolization of pseudoaneurysm. Successful thrombosis of aneurysm with no distal flow. A. Early phase. B. Late phase.

During the subsequent angiogram, vascular supply from the ileocolic branch and ileojejunal branch surrounding the ileal branch aneurysm was found indicating adequate collateral flow for embolization. The decision was made to embolize the aneurysm sac itself after prolonged attempts of trying to embolize both proximal and distal branches of the vessel. Two 8 mm, 14 cm length Nestor coils, and three additional 6 mm, 15 cm length Nestor coils were advanced into the aneurysm. This resulted in successful thrombosis of the aneurysm with no distal flow seen here on both the early phase and late phase seen in Figure 3A,B, respectively. The patient was asymptomatic in his early initial phase and the patient was followed up in the clinic of the primary care physician at seven days and four weeks postprocedure when he was found to be doing well.

## Discussion

Visceral artery aneurysm and VAPA of the splanchnic arteries are an uncommon incidental finding found in relatively asymptomatic clinical settings. Visceral artery true aneurysms contain all three layers of the blood vessel wall, compared to pseudoaneurysms which occur due to damage to the blood vessel wall and subsequent accumulation of blood between the tunica media and tunica adventitia. Generally, VAA is much more common than VAPA. Visceral pseudoaneurysms, however, occur more frequently in gastroduodenal and superior mesenteric arteries compared to true aneurysm [3]. The most common way a VAPA is diagnosed is through an incidental finding on imaging due to their asymptomatic nature. However, VAPA presenting from the superior mesenteric artery is usually symptomatic. Habib et al. described a systematic review of literature in which they found gastrointestinal hemorrhage as the most common presenting symptom followed by abdominal pain [4]. The wide breadth of gastrointestinal (GI) conditions that may present with vague abdominal pain, hematochezia, hematemesis, melena, and other hemorrhagic symptoms further qualifies the necessity to discuss rare VAPA. Superior mesenteric artery (SMA) pseudoaneurysms that have ruptured are associated with a 37% mortality rate [5]. 

Pseudoaneurysms can have a plethora of etiologies. SMA pseudoaneurysms, specifically, are most frequently caused by inflammation from pancreatitis or other etiologies, or trauma [5]. Trauma can be any variety of iatrogenic causes from surgery or angiography, to accidents or penetrating trauma.

 Currently, pseudoaneurysms are most commonly identified incidentally on CT scans of asymptomatic patients. Initial diagnostic steps for VAPA should include CT followed by angiography. Angiography allows for more specific localization of the suspected pseudoaneurysm as well as confirmation of collateral flow. Prompt diagnosis and management should be the top priority in anyone suspected of a VAPA because of the high rate of rupture and hemorrhage. According to a study by Pitton et al. pseudoaneurysms are far more likely than true aneurysms to rupture (76.3% vs. 3.1%) [6]. Thus, VAPA should be managed immediately either surgically or with endovascular techniques.

Surgical technique involves an open technique in which the aneurysm sac is excised and vascular flow is regained. Indications for surgery of aneurysms include those patients that are symptomatic, aneurysms in pregnant women, and asymptomatic aneurysms greater than 2 cm. Elective surgeries for aneurysms have a mortality rate that is around 1.5% according to a study done by Pulli et al. [7]. Less invasive techniques of repair have emerged in the modern era such as endovascular coiling, stent placement, and thrombin injections. The basic principles governing endovascular technique are the exclusion of the aneurysmal sac, to bypass the aneurysmal sac, or to ligate it. Often the specific less invasive technique depends on the ease of access to the pseudoaneurysm itself, the size of the VAPA sac, and the exact location of the VAPA. In this case, based on CT scan and intraoperative angiography, we chose to use transcatheter coil embolization. A study done by Tulsyan et al. showed that the success rate of endovascular coil embolization approached 96% [8]. The traditional approach to coil embolization includes embolizing both the proximal and distal arteries. The cornerstone behind this approach is adequate collateral blood supply to prevent ischemia. In our case, the adequate collateral blood supply was seen; however, embolization of proximal and distal branches was not successful. The next best option in this approach is to embolize the sac itself, which is the route that was taken in this case. Complications from endovascular techniques include rupture of the sac itself, ischemia distal to the pseudoaneurysm sac, and possible arterial flow to the pseudoaneurysm after delayed reconstitution of blood (postembolization syndrome). A study done by Piffaretti et al. found that postembolization syndrome occurred in five of the 15 patients treated with endovascular coiling [9]. Another study by Tulsyan et al. showed that only three patients of the 48 developed postembolization syndrome [8].

 New emerging technology in the management of aneurysms is a flow diverting stent. These are unique in that flow to collateral vessels will be preserved, as is not the case in other techniques. Flow-diverting stents are a type of flow modulator, meaning a stent with a certain porosity will be placed in the parent vessel allowing minimal flow to the sac itself. Minimal flow into the sac itself will then cause stagnation and eventual thrombosis of the sac, as seen in almost 88% of aneurysms using this method by Kallmes et al. [10]. It has been shown by Liou et al. that turbulent flow into the aneurysmal sac may decrease to 5% of the parent vessel in those flow-diverting stents with a porosity of 40% or less [11]. One of the bigger studies in recent years was done by Ruffino and Rabbia in which they showed that 87% of patients treated with flow diversion stents had patency at one month and that same percentage had sac thrombosis at one month [12].

## Conclusions

Pseudoaneurysms are a rare entity seen in clinical practice. With the advent of newer and more accurate technologies, more of these will be incidentally found. Prompt diagnosis and immediate management remain the mainstay of the workup for all identified pseudoaneurysms. The case itself identified abnormal symptomatology associated with the diagnosis as well as a different approach to endovascular therapy of pseudoaneurysms. We hope this case aids clinicians in the future care of patients with visceral artery aneurysms and pseudoaneurysms.
